# The efficacy of ultrasound-guided anterior quadratus lumborum block for pain management following lumbar spinal surgery: a randomized controlled trial

**DOI:** 10.1186/s12871-022-01943-8

**Published:** 2022-12-19

**Authors:** Selcuk Alver, Ciftci Bahadir, Ali Can Tahta, Ahmet Cetinkal, Birzat Emre Gölboyu, Cem Erdogan, Mursel Ekinci

**Affiliations:** 1 Department of Anesthesiology and Reanimation, Istanbul Medipol University, Istanbul, Turkey; 2 Department of Neurosurgery, Istanbul Medipol University, Istanbul, Turkey; 3 Department of Anesthesiology, Katip Çelebi University, Izmir, Turkey; 4 Department of Anesthesiology and Reanimation, Bursa State Hospital, Bursa, Turkey

**Keywords:** Quadratus lumborum block, Lumbar disc herniation surgery, Postoperative pain, Regional anesthesia

## Abstract

**Background:**

Quadratus lumborum block (QLB) is a fascial plane block. There is no randomized study on the efficacy of QLB for lumbar surgery. We evaluated the efficacy of QLB for postoperative pain management and patient satisfaction after lumbar disc herniation surgery (LDHS).

**Methods:**

Sixty patients with ASA score I-II planned for LDHS under general anesthesia were included. We allocated the patients into two groups: the QLB group (*n* = 30) or the control group (*n* = 30). QLB was performed with 30 ml 0.25% bupivacaine in the QLB group. Paracetamol 1 g IV 3 × 1 was ordered to the patients at the postoperative period. If the NRS score was ≥ 4, 1 mg/ kg tramadol IV was administered as rescue analgesia.

**Results:**

There was a reduction in the median static NRS at 0 h and 2 h with QLB compared to the control group (*p* < 0.05). There was no difference in the resting NRS at any other time point up to 24 h. The median dynamic NRS was significantly lower at 0, 2, 4, 8, and 16 h in the QLB group (*p* < 0.05). The need for rescue analgesia was significantly lower in the QLB group. The incidence of nausea was significantly higher in the control group. The postoperative patient satisfaction was significantly higher in the QLB group (*p* < 0.05).

**Conclusion:**

We found that the QLB is effective for pain control following LDHS.

## Introduction

Lumbar disc surgery is a common procedure performed for leg and lower back pain. It commonly provides satisfactory results for most patients [1, 2]. However, pain after surgery is a major problem. In these patients, severe pain may occur in the postoperative period, especially in the surgical area. Meta-analyses have shown that a consistent proportion of patients experience short-term back or leg pain after lumbar disc herniation surgery (LDHS). Though LDHS is a minimally invasive surgery, patients may suffer from moderate-to-severe pain afterward [3–5]. Effective postoperative pain management supports early mobilization and shorter hospital stays, and may thus reduce the likelihood of complications such as infection and thromboembolism. Thus, an effective acute postoperative pain management strategy has critical importance.

Ultrasound (US)-guided regional anesthesia methods have recently become popular in daily anesthesia practice, and they have great potential to support effective postoperative pain management [6, 7]. A US-guided quadratus lumborum block (QLB) is a posterior abdominal wall fascial plane block first described by Blanco [8], where local anesthetic (LA) is administered around the QL muscle [9, 10]. LA spreads to the middle layer of the thoracolumbar fascia (TLF) in a triangular region named the lumbar interfascial triangle (LIFT) [8–14]. The LIFT is related to the thoracic paravertebral space [13, 14]. Given its mechanism of action, the QLB may be an alternative analgesic technique for spinal surgeries. Yet, to our knowledge, no randomized study had yet been carried out on the efficacy of QLB for lumbar surgery. So, in this prospective randomized study, we evaluated the efficacy of US-guided QLB for postoperative pain management and patient satisfaction after LDHS.

## Methods

**Fig. 1:**
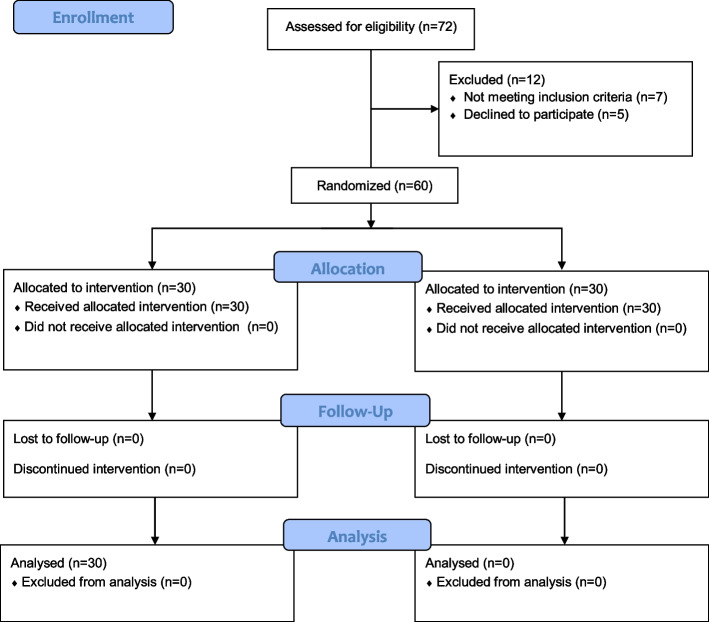
CONSORT flow diagram of the study

Ethical approval for this randomized prospective study was provided by the Istanbul Medipol University Ethics and Research Committee (21.04.2021, decision no. 429). After approval, the trial was added to a clinical trial registry (NCT04981301. First registration date is 28/07/2021). American Society of Anesthesiologists status 1–2 patients aged between 18 and 65 years scheduled for elective single-level lumbar discectomy and hemilaminectomy surgery under general anesthesia were enrolled in the trial. The study procedure was explained to the patients and their written informed consent was obtained. When enrolling the patients, we followed the steps of the Consolidated Standards of Reporting Trials (CONSORT) flow diagram (Fig. 1). The study was conducted at the Medipol Mega Hospital Complex from August to December 2021.

The exclusion criteria were coagulopathy, anticoagulant treatment, history of local anesthetics, allergy, localized infection in the block procedure area, pregnancy or breastfeeding, inability to understand or use the verbal-rated pain-scoring system, and refusal to participate in the study. Using a computerized randomization program, we allocated the patients to one of two groups: the QLB group (*n* = 30) or the control group (*n* = 30).

### General anesthesia

After arriving in the operation room, the patients were monitored with electrocardiography, non-invasive blood pressure, and pulse oximetry. Propofol (2–2.5 mg/kg), fentanyl (1–1.5 µg/kg), and rocuronium bromide (0.6 mg/kg) IV was used for classical anesthesia induction. The patients were placed in the prone position following intubation for surgery. Sevoflurane with a mixture of oxygen + fresh air, and remifentanil (0.5 – 1 µg/kg/min) infusion was used for anesthesia maintenance. All patients underwent unilateral single-level lumbar microdiscectomy/hemilaminectomy surgery by the same surgical team using the same technique. The wound size was 12 mm routinely. Paracetamol (1 g) and a dose of 100 mg tramadol were intravenously administered to all patients 30 min before the end of the surgery for postoperative analgesia. Moreover, the patients were given 4 mg ondansetron to prevent nausea and vomiting. The QLB was performed at the end of the surgery before extubation. In the control group, a dose of 0.25% bupivacaine (30 ml) was injected for skin analgesia by the surgeon around the surgical area. Patients with sufficient spontaneous respiration were extubated and then transferred to the post-anesthesia care unit (PACU).

### QLB technique

**Fig. 2:**
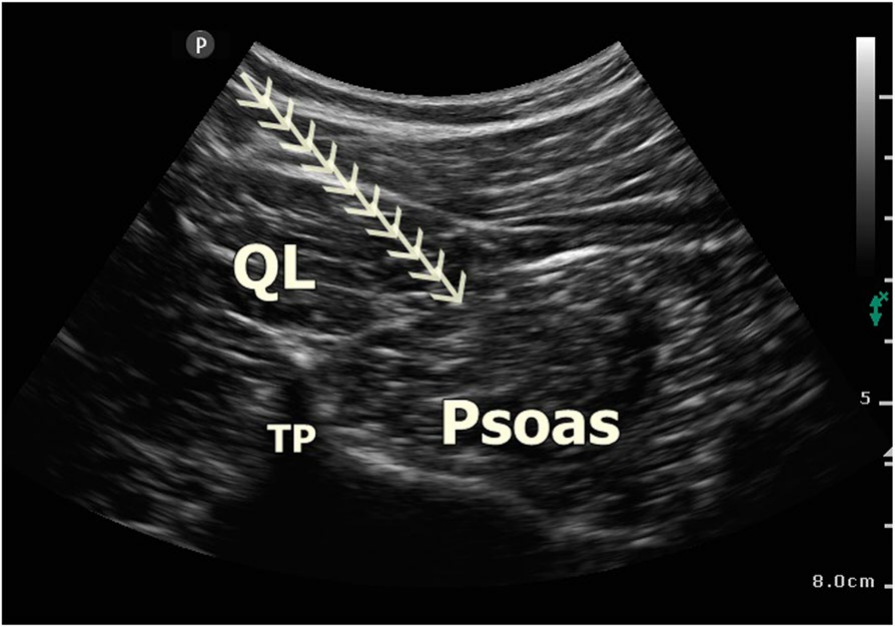
Sonographic visualization of anterior QLB. Quadratus lumborum muscle, psoas muscle, and transverse process are seen. The arrows indicate the needle direction. *QL* quadratus lumborum, *TP* transverse process

The QLB was performed using US (Vivid Q, GE Healthcare, USA) at the end of the surgery before extubation, with patients in the prone position. Under aseptic conditions, the convex transducer was covered with a sterile sheath. The transducer was placed just above the iliac crest and moved cranially to visualize the three abdominal muscles, psoas muscle, and the QL muscle (Fig. 2). A 22 g, 100 mm block needle (Braun Stimuplex Ultra 360, Germany) was employed. Using a posterior-to-anterior trajectory, the needle was inserted taking an in-plane approach through the QL muscle (anterior QLB). The needle tip was placed on the anterior border of the QL muscle (between the QL and psoas muscles) and 5 ml saline was injected into the fascial plane. After correction of the block region, 15 ml 0.25% bupivacaine was injected here. The same process was performed on the opposite side (in total, injecting 30 ml local anesthetic).

### Standard postoperative analgesia protocol, measurement of pain, and outcomes

In the postoperative period, 1 g paracetamol was administered intravenously every 8 h. Patients were evaluated by a pain nurse who did not know how the patients were grouped. The patients were blinded with regards to the treatment received. Their postoperative pain was assessed using the 11-point Numerical Rating Scale (NRS), which ranges from 0 (meaning “no pain”) to 10 (meaning “worst pain imaginable”). The NRS scores were recorded at rest (static) and during mobilization (dynamic) at 0 (PACU), 2, 4, 8, 16, and 24 h. If the NRS score was ≥ 4, 1 mg/ kg tramadol IV was administered as rescue analgesia. Adverse effects such as nausea, vomiting, and itching were recorded. Patient satisfaction was assessed with the Likert scale.

The primary outcome was the dynamic pain score in the PACU (during a motor power assessment test) postoperatively. Pain scores were assessed using the NRS.

The secondary outcomes were the need for rescue analgesia, adverse effects, and the measurement of patient satisfaction, as recorded during the initial 24-h postoperative period. The need for rescue analgesia was evaluated as “used” or “not used” (yes/no). The incidence of nausea/vomiting/itching was assessed as “yes” or “no.” Patient satisfaction related to postoperative analgesia was evaluated using a seven-item Likert scale (extremely dissatisfied, mostly dissatisfied, somewhat dissatisfied, neutral, somewhat satisfied, mostly satisfied, extremely satisfied).

### Statistical analysis and sample size calculation

A preliminary study was performed on eight patients from each group in our clinic. We considered a reduction in mean pain scores (dynamic NRS in the PACU) by two points to be clinically meaningful and important. For a two-sided test, assuming a mean (SD) pain score of 4.5 (2) in the control group, a sample size of 29 per group had a power of 85% to detect a statistically significant difference in mean pain scores of two or more using Student’s t-test, with the alpha set to 0.05 and the sample size estimated using the G*Power 3 analysis program (Heinrich-Heine Universitat, Dusseldorf, Germany).

The shape of the distribution of the variables in this study was assessed using the Shapiro–Wilk test, to determine whether the observations were normal or skewed. In cases where the test results indicated that the data were normally distributed, the data were presented with the mean ± SD and analyzed using an independent samples t-test to compare groupwise differences in the outcome parameters. Meanwhile, we presented continuous data that yielded a non-parametric dispersion with the median and IQR, and we analyzed these using the Mann–Whitney U test to observe the groupwise differences. Statistical analyses were conducted using SPSS V.25 (SPSS, Chicago, IL, USA).

## Results

We recruited 60 patients, with 30 allocated randomly to each group during the study period. Demographic data are shown in Table 1, where it can be seen that there were no differences between the groups, or complications or adverse events related to the block procedure.

**Table 1:** Comparison of demographic data and duration times of surgery and anesthesia between QLB and Control group

	**Group QLB** **(n:30)**	**Group Control** **(n:30)**
Gender (M/F)	16/14	16/14
Age (years)	50.5 ± 10	45.3 ± 10.8
Weight (kg)	79.7 ± 11	76,6 ± 10.4
Height (cm)	167 ± 10	167 ± 8
ASA I/II	11/19	15/15
Duration of surgery (min)	82.3 ± 17	89,1 ± 13.3
Duration of anesthesia (min)	101.6 ± 18	107,7 ± 12.3

Values are expressed mean ± standart deviation or number

*kg* kilogram, *cm* centimeter, *M* male, *F* female, *min* minutes, *ASA* American Society of Anesthesiologist

We found a reduction in the median (IQR [range]) static NRS scores at 0 h and 2 h with QLB compared to the control group (2 [0–2] vs. 2 [2, 3], *p* = 0.001 and 2 [1,2] vs. 2 [2,3], *p* = 0.003, respectively; *p* = 0.001 and *p* = 0.003, respectively). There was no difference in the resting NRS scores at any other time point up to 24 h (Table 2). The median (IQR [range]) dynamic NRS scores were significantly lower at 0, 2, 4, 8, and 16 h in the QLB compared to the control group. There was no difference in the dynamic NRS scores at 24 h between groups (Table 2).

**Table 2:** Comparison of the Numerical Rating Scale scores between QLB and Control group

**NRS Static**			
**Hour**	**Group QLB** **Median (IQR)** **(n:30)**	**Group Control** **Median (IQR)** **(n:30)**	***P***
0	2 (0–2)	2 (2–3)	**0.001**
**2**	2 (1–2)	2 (2–3)	**0.003**
**4**	1 (0.75–2)	2 (1–2)	0.190
**8**	1 (0–2)	1 (1–2)	0.090
**16**	1 (0.25–1)	1 (1–1)	0.139
**24**	0 (0–1)	1 (0–1)	0.926
**NRS Dynamic**			
**Hour**			
**0**	3 (1–3)	4 (3–4)	**0.002**
**2**	2 (2–3)	3 (2–3)	**0.011**
**4**	2 (1–3)	3 (2–3)	**0.025**
**8**	1 (1–2)	2 (2–3)	**0.008**
**16**	1 (0–2)	2 (2–2)	**0.015**
**24**	0 (0–1)	1 (0–1)	0.390

Values are expressed mean ± standart deviation, NRS: Numerical Rating Scale,

*P* value is obtained with Mann–Whitney U test. Median (IQR)

The need for rescue analgesia was significantly lower in the QLB group (9 QLB patients vs. 19 control patients, *p* = 0.019). The incidence of nausea was significantly higher in the control group compared to QLB (13 control patients vs. 5 QLB patients, *p* = 0.047). There were no differences in terms of vomiting or itching (*p* = 1 and *p* = 0.23, respectively). The postoperative patient satisfaction (Likert scale) was significantly higher in the QLB group compared to the control (*p* = 0.001, Table 3).

**Table 3:** The Comparison of incidence of adverse effects, patients satisfaction, and the need of rescue analgesia between QLB and Control group

	**Group QLB (n:30)**	**Group Control** **(n:30)**	**P**
Nausea (Y/N)	5/25	13/17	**0.047†**
Vomiting (Y/N)	3/17	4/26	1†
Itching (Y/N)	5/25	10/20	0.23†
Patient satisfaction (Likert)	6(4–6)	3(3–5)	0.001*
The need of rescue analgesia (Y/N)	9/21	19/11	**0.019†**

^†^*P* value is obtained with Pearson’s χ2 test (n)

^*^P value is obtained with Mann–Whitney U test (Median (IQR))

*Y* Yes, *N* No

## Discussion

To our knowledge, this study is the first randomized, prospective, controlled study to have evaluated the efficacy of QLB for postoperative analgesia management after LDHS. According to our results, QLB provided pain relief after surgery; compared to the control group, QLB decreased patients’ pain scores (NRS, especially during movement) and reduced their need for rescue analgesia. Furthermore, patient satisfaction was higher in the QLB group.

The QLB was first described by Blanco, and since then, it has been modified and different approaches with different needle trajectories described [8–10]. The TLF is a fibrous compound of fascial tissue that surrounds the QL muscle. The TLF is a complex structure, comprising several layers, which separate the paraspinal muscles from the posterior abdominal wall muscles, QL, and psoas major [11–14]. The LIFT is formed in the triangular anatomical structure formed by TLF [9, 11–13]. The target of local anesthesia during the QLB is the LIFT [8, 9]. By this proximity between TLF and lumbar region, QLB may provide analgesia after spine surgeries.

There are three approaches to the QLB in the current literature [9–12]. The target of lateral QLB (QLB 1) is the anterolateral side of the QL with an anteroposterior trajectory. Posterior QLB (QLB 2) targets the posterolateral side of the QL muscle. The target injection point of the anterior QLB (QLB 3/transmuscular QLB) is between the QL and psoas muscles with a posteroanterior trajectory [9, 11, 12]. We performed anterior QLBs in our study. In the literature, randomized controlled studies can be found that investigated the efficacy of QLB for several abdominal surgeries [8, 15–18], but none address lumbar surgeries. An anterior QLB has been reported to spread to the paravertebral space, involving somatic nerves and the sympathetic trunk at the T9–10 level in cadavers [19]. Our results show that, QLB is effective for such spine surgeries.

The QLB is a safe, effective fascial plane block that is easy to apply. According to our results, an anterior QLB may be a good analgesic option for lumbar spine surgery. We followed the patients in this study for 24 h and recorded lower pain scores and less of a need for rescue analgesia in the QLB group. The median duration of analgesia after the QLB exceeded 16 h, but that duration may have depended on the volume we used (30 ml in total, i.e., 15 ml for each side). In the literature, the volume used for the QLB is often 40 ml. Since fascial plane blocks are volume-dependent, the analgesic period may have been longer if we used a higher volume than 30 ml. Though our study did not focus on the dermatome level, the pain relief in the QLB group showed that the QLB provided analgesia after LDHS. Though the QLB is safe, potential unintended complications such as abdominal organ injury and hematoma should be kept in mind [8, 20]. In addition, with multimodal analgesic regimens including a non-steroidal anti-inflammatory drug and an opioid, usually provides pain control and saves the time spent. Clinicians may prefer regional anesthesia techniques according to their experiences. We aimed to present a novel option for lumbar surgery, QLB block. On the other hand, another techniques may be preferred for pain management after LDHS. Lumbar erector spinae plane block (ESPB) is one of these techniques. ESPB is performed deeply the plane of erector spinae muscle above the transvers process. In the literature it has been reported that ESPB provides pain relief after lumbar surgeries [21–23]. Another option is modified-thoracolumbar interfascial plane block (M-TLIP). M-TLIP block is performed between iliocostalis and longissimus muscles (parts of erector spinae muscles). It has been reported that M-TLIP block provides analgesia after lumbar operations [23, 24].

### Limitations

Our study has some limitations that must be noted before we conclude. Firstly, we used a single total volume of 30 ml local anesthetic, though different results may be achieved with different volumes. Secondly, we used only an anterior approach to the QLB, creating a need to investigate other approaches to the QLB for lumbar spine surgery. Lastly, we did not evaluate the dermatome level after the block’s application. In this research, we evaluated the efficacy of the QLB based on the patients’ pain scores and need for rescue analgesia, but in the future, larger studies are needed to corroborate our findings on the efficacy of the QLB after LDHS.

## Conclusion

In conclusion, according to the results of our study, an anterior QLB lowers patients’ postoperative pain scores and reduces the need for rescue analgesia. Overall, we found that the QLB is effective for pain control following LDHS.

## Data Availability

The datasets used and/or analysed during the current study are available from the corresponding author on reasonable request. The email of corresponding author is bciftci@medipol.edu.tr.

## Abbreviations

CONSORT: Consolidated Standards of Reporting Trials
LA: Local anesthetic
LDHS: Lumbar disc herniation surgery
LIFT: Lumbar interfascial triangle
NRS: Numerical Rating Scale
PACU: Post-anesthesia care unit
QLB: Quadratus lumborum block
TLF: Thoracolumbar fascia
US: Ultrasound
